# Weaning and extubation from neonatal mechanical ventilation: an evidenced-based review

**DOI:** 10.1186/s12890-022-02223-4

**Published:** 2022-11-16

**Authors:** Razieh Sangsari, Maryam Saeedi, Marzieh Maddah, Kayvan Mirnia, Jay P. Goldsmith

**Affiliations:** 1 Department of Pediatrics, Division of Neonatology, Faculty of Medicine, Tehran University of Medical Sciences, Children’s Medical Center, Pediatric Center of Excellence, Tehran, Iran; 2 Department of Pediatrics, Division of Neonatology, Faculty of Medicine, Shahid Beheshti University of Medical Sciences, Shohadaye Tajrish Hospital, Tehran, Iran; 3 Division of Newborn Medicine, Tulane University School of Medicine, New Orleans, Louisiana, USA

**Keywords:** Non-invasive ventilation, Infant, Airway extubation, Ventilator weaning

## Abstract

Mechanical ventilation is a lifesaving treatment used to treat critical neonatal patients. It facilitates gas exchange, oxygenation, and CO2 removal. Despite advances in non-invasive ventilatory support methods in neonates, invasive ventilation (i.e., ventilation via an endotracheal tube) is still a standard treatment in NICUs. This ventilation approach may cause injury despite its advantages, especially in preterm neonates. Therefore, it is recommended that neonatologists consider weaning neonates from invasive mechanical ventilation as soon as possible. This review examines the steps required for the neonate's appropriate weaning and safe extubation from mechanical ventilation.

## Background

Mechanical ventilation is a lifesaving treatment used in critical neonatal patients. It is indicated when the patient's spontaneous ventilation is insufficient to sustain life. Mechanical ventilation expedites and facilitates gas exchange, oxygenation, and CO2 removal. Despite advances in non-invasive ventilatory support methods, intubation and mechanical ventilation are still common treatments in NICUs.

A large cohort study in 2005 found that 89% of the extremely low birth weight (ELBW) neonates were treated with mechanical ventilation in the early days of life, and 95% of the neonates that survived in this group had a history of invasive (i.e., via an endotracheal tube) mechanical ventilation during hospitalization [1]. Another cohort study reported that 74% of the neonates born before 28weeks of gestational age were intubated and received surfactants during hospitalization [2].

This treatment method, despite its advantages, may cause injury. Reported complications include ventilator-induced lung injury (VILI) [3], ventilator-associated pneumonia (VAP), tracheal injuries, and neurodevelopmental impairment [4]. Therefore, it is now recommended that neonatologists consider weaning premature neonates from mechanical ventilation as soon as possible [5].

About 20 years ago, the American College of Chest Physicians recommended protocols for weaning adult patients from respiratory support machines. These guidelines were prepared according to randomized controlled trials (RCTs) that showed improved outcomes for adults managed using these protocols [6]. However, it is inappropriate to apply adult data to term and premature neonates due to the immature control of ventilation, unique lung physiology, ventilation mechanics, and types of lung disease found in newborns.

In 2009, Hermeto, in an observational study conducted in Canada, showed protocols for managing an intubated neonate with a birth weight of 1250 g or less and using objective criteria for weaning, extubation, and reintubation resulted in a marked improvement in outcomes. Protocol use led to earlier extubation, lower extubation failure rates, and reduced time on mechanical ventilation in days [7]. In 2011, a systematic review by Blackwood et al. showed that using protocols for weaning neonates from mechanical ventilation reduced the length of hospital stay and length of time on mechanical ventilation [8]. However, Bas Bol et al. conducted a review in 2020 and found that weaning protocols in neonates were used in a small percentage of patients and had contradictory results [9]. In general, these studies have supported the need for protocols in NICUs; however, more RCTs are required to evaluate the effects on preterm neonates. Although there are several mechanical ventilation strategies for neonates, new neonatal ventilator modalities, such as auto-weaning from invasive ventilation, make it challenging to formulate a universal protocol that will serve all patients and providers. Nonetheless, evidence-based medicine can be used to design some guidelines and protocols for specific groups of patients.

## Main text

### Ventilation modes and strategies

With rapid technological advances and new ventilation modes for neonates, neonatologists can choose various options. However, the most widely used ventilatory modes for treating neonates are pressure-targeted (assist-control [A.C.] and synchronized intermittent mechanical ventilation [SIMV] ), volume-targeted, and high-frequency ventilation.

#### Pressure targeted ventilation

This method has been used to treat respiratory failure in preterm neonates for decades. The basic principle of pressure-targeted ventilation is to create enough pressure to open the airway, overcome the respiratory tract and lung parenchyma's resistance, and finally, result in the gas flow to the alveoli. The gas volume that reaches the alveoli depends on lung compliance, inspiratory pressure and time, flow, and synchronization of the ventilator with the baby's spontaneous breaths [10].

In this type of mechanical ventilation, weaning starts with reducing peak inspiratory pressure (PIP) and rate. The amount of reduction in PIP and rate depends on lung compliance. The respiratory rate usually decreases gradually to 10-15 breaths/min; however, many new guidelines support extubation at higher respiratory rates (> 20 breaths per minute) if the respiratory drive is good. PIP may be reduced simultaneously, but it should not be reduced to the extent that tidal volume falls below 4-5 ml/kg unless spontaneous ventilation supplements the minute volume.

In SIMV, spontaneous breaths beyond the SIMV rate are not supported, which may result in unwanted tidal volumes and increased work of breathing. This condition is more common during weaning, i.e., when the SIMV rate decreases and unsupported spontaneous breaths increases. It is more critical in ELBW neonates with narrow ETTs because resistance is inversely proportional to the 4^th^ power of the internal radius of the ETT (Poisuille's Law). The high resistance of airways and small diameter ETTs, poor muscle power, and excessive chest wall compliance in neonates results in small ineffective tidal volumes, increasing dead space, and reduced adequate alveolar ventilation.

Despite these considerations, many physicians still prefer SIMV for weaning neonates; based on this traditional belief, lower respiratory rates in this mode are less harmful and that decreasing the mechanical rate will strengthen the spontaneous muscle activity for breathing. Nonetheless, it has been shown that excess tidal volume causes overexpansion and direct lung injury without considering the pressure required to produce them. Therefore, although more breaths are delivered in the A.C. mode, they may be less harmful since the tidal volume is 33% less. Another controversy is that supporting each breath wastes the opportunity to strengthen the respiratory muscles, which fails to consider the complex neonate-ventilator interaction during synchronized ventilatory support. In synchronized ventilation, tidal volume is produced due to a combination of the patient's respiratory effort (negative intrapleural pressure) and a positive pressure generated by the ventilator. This combined respiratory effort (ventilator pushing-baby pulling) produces a transpulmonary pressure that, along with the respiratory system compliance, results in a given tidal volume. However, depending on the synchronization method (flow, Graseby capsule, neurally adjusted ventilatory assistance [NAVA], etc.), only 50-80% of breaths are truly synchronized; in a time cycle, the inspiration ends, and expiration begins, even if the neonate is still in the inspiration phase, so only the inspiration phase is synchronized with the patient.

As the PIP decreases during weaning, the neonate gradually takes on more breathing work, which may strengthen the respiratory muscles. Moreover, the ventilator pressure is reduced when it overcomes the added resistance of the ETT and circuit, indicating readiness for extubation. At this point in small preterm neonates, it is better to add additional support or Pressure Support (PS) to SIMV when the rate falls below 30/min, which will reduce the work of breathing and make weaning faster.

Some studies have recommended NAVA mode in cases with difficult weaning. Electrical signals from the diaphragm are detected in the NAVA system triggering respiratory assistance, which is applied simultaneously with the diaphragmatic movement. The synchronized respiratory support with the infant's spontaneous efforts may facilitate weaning in these neonates [11].

#### Volume Targeted Ventilation (VTV)

When tidal volume instead of pressure is the primary control variable with conventional mechanical ventilation, pressure reduction occurs automatically in response to the neonate's compliance improvement and increased respiratory effort. This decrease in respiratory support occurs in a real-time and continuous manner instead of reacting to intermittent ABG sampling and changing the ventilator settings, and therefore, it may accelerate weaning.

Klingenberg et al., in a Cochrane meta-analysis regarding volume guarantee and VTV in infants of less than 44 weeks post-conceptual age, showed that the volume-targeted modes markedly reduced the duration of mechanical ventilation compared to pressure-targeted modes. Meta-analysis performed on the skewed data gave a mathematical mean deviation of -1.35 days (95% CI -1.83 to -0.86) of reduced duration of ventilation using VTV [12].

VTV is the most widely used invasive ventilation mode in premature neonates due to its apparent benefits in reducing mortality, bronchopulmonary dysplasia (BPD), intraventricular hemorrhage (IVH), and length of time on mechanical ventilation. The tidal volume setting can vary between 4 and 12 ml/kg in different disease states due to differences in underlying pathology, lung compliance, and resistance [10, 13], although the higher tidal volumes are associated with increased morbidities.

As noted above, in this ventilatory mode, the ventilatory support is reduced automatically, and the required pressure to achieve the set tidal volume is decreased as pulmonary compliance improves. However, if the target tidal volume is reduced below average physiologic values, the work of breathing increases disproportionately.

Long-term ventilator-dependent neonates need larger tidal volumes over time secondary to increased anatomic and/or physiologic dead space for multiple reasons, including acquired tracheomegaly and segmental atelectasis. Moreover, considering the heterogeneous aeration of the lungs, the tidal volume should not generally fall below four to five ml/kg during weaning to prevent increasing the work of breathing [14].

Ventilation with a high tidal volume may cause alveolar injury and volutrauma. On the other hand, delaying extubation until a very low tidal volume causes atelectasis and alveolar collapse, affects the extubation outcome and increases the chance of extubation failure. Gupta et al. have shown that neonates weaned from mechanical ventilation successfully had higher tidal volumes before extubation, and the amount of tidal volume at the time of extubation could predict extubation success with moderate sensitivity and specificity [15].

Desirable tidal volumes for weaning are still debated and depend on numerous factors such as gestational age and disease state. During weaning, using low-volume settings in VTV while decreasing the ventilator rate increases the work of breathing; therefore, a tidal volume of 5-7 ml/kg is recommended [10].

Although extubation failure depends on several parameters, one of which is tidal volume, a lower than normal tidal volume before extubation has been associated with an increase in the failure rate [14].

#### High-Frequency Ventilation (HFV)

Although many physicians change the mode from HFV to conventional ventilation in the weaning process, it is possible to extubate the patient directly from HFV.

Clark's study compared the two ventilatory strategies using HFOV and conventional ventilation. The eligible population was premature neonates weighing less than 1751 grams birth weight with respiratory distress syndrome (RDS). The study revealed a lower chance of BPD in neonates who remained on high-frequency oscillatory ventilation (HFOV) until extubation compared to neonates who were switched to SIMV after 72 hours of HFOV [16]. Similarly, Courtney et al. showed a lower chance of BPD and a shorter length of days on mechanical ventilation in neonates treated with HFOV in the first two weeks after birth until extubation [17]; however, a Cochrane review did not confirm these findings [18].

### Assessment of extubation readiness

Extubation failure (defined as the need for reintubation in the first 2-7 days after extubation) has been reported as high as 10-80% in different populations of VLBW infants [19]. Different tools, such as estimators and machine learning approaches, have been used to evaluate extubation readiness in neonates in recent years. Despite advances in the mechanical ventilation of neonates, it is still a challenge to identify whether a neonate is ready for extubation. Meticulous serial evaluations of readiness for extubation are essential in weaning neonates from mechanical ventilation since prolonged mechanical ventilation is harmful, but early extubation may be associated with respiratory failure, reintubation, and sometimes tragic complications.

Prediction of the extubation outcome is a complex process that depends on several parameters, such as sufficient neural signals and neuromuscular synapses, the functional capacity of respiratory muscles, and the primary pathology of the lung. Univariate and multivariate models have been designed to evaluate extubation readiness; however, no accepted model has yet been developed [19]. The most critical aspect of discontinuing respiratory support is improving the underlying cause of respiratory failure and its complications. Once an adequate gas exchange is achieved with a low PEEP and FiO2 in a hemodynamically stable neonate and respiratory drive shifts towards spontaneous breathing, the neonate is ready to discontinue invasive mechanical ventilation and extubation.

### Clinical assessment

Different methods and techniques have been used for the clinical assessment of neonates before and following extubation. Recent cohort studies have shown that extubated neonates successfully were heavier, more mature, and required less oxygen within the first 24 hours of birth and before extubation than the failed cases. Moreover, the successful extubation group had lower PCO2 levels, mean airway pressure (MAP), and higher pH values before extubation [20, 21].

Several studies assessed the Tension-Time Index (TTI), which evaluates respiratory muscle strength, as a tool for determining extubation readiness. In a study of 20 neonates born at 24-39 weeks' gestation receiving mechanical ventilation in 2011, Currie found that the TTI of the diaphragm and respiratory muscles predicted extubation success with high sensitivity and specificity. The results also showed diaphragmatic dysfunction in neonates with extubation failure [22]. However, subsequent studies showed that TTI is less sensitive than gestational age and birth weight in predicting extubation readiness, and its use was not recommended in clinical practice [21, 23].

Minimal ventilator settings before extubation is one of the parameters used to signal the readiness of neonates for extubation; however, there is currently no consensus for the definition of minimal settings in preterm neonates. In the APEX cohort study, neonates were extubated from different settings and parameters, including MAP of 5-14 cmH2O, FiO2 requirements from 21% to 53%, and PCO2 levels from 22 to 69 mmHg [23]; no significant difference was observed in the success of extubation. Therefore, it seems impractical to determine specific parameters as the appropriate minimal settings to attempt extubation [24]. Some physicians prefer early extubation within the first days of life, but others prefer to delay extubation until the neonate becomes more mature and has achieved positive nutritional nitrogen balance before attempting extubation. However, retrospective studies have shown a significant relationship between early extubation and improved outcome [25, 26]. In some studies, delayed extubation beyond 3-7 days after birth was associated with an increased risk of BPD and a worse outcome [27]. Overall there is no definite evidence for determining an optimal time for extubation.

### Spontaneous breathing trial

Spontaneous breathing trials (SBT) of 30-120 minutes for assessing readiness for extubation have proved effective in adults and are evidence-based [28]. Several studies have evaluated this method in neonates and found that neonates who underwent SBT were extubated faster than those only assessed clinically. According to these studies, SBT has high sensitivity and positive predictive value but low specificity and negative predictive value in evaluating the neonates' readiness for extubation. Therefore, despite its low costs and availability, SBT cannot be considered a test with accurate results in assessing the neonates' readiness for extubation [24, 29–31]. The ability of SBT to predict success may be due to the increased resistance and dead space of the endotracheal tubes in small premature on CPAP, which increases the work of breathing. Those that can tolerate this added work will have a greater chance of being successfully extubated. Moreover, the time and level of appropriate post-extubation CPAP and the failure criteria are unclear, and more extensive studies are needed. Other techniques, such as measuring minute ventilation on CPAP for 10 minutes and comparing it to the minute ventilation on mechanical ventilation before the CPAP trial, have reported increased extubation success [32].

### Autonomic nervous system (ANS) function

Evaluating the ANS function during weaning can provide valuable information about physiopathological imbalance. A lack of heart rate variability (HRV) in preterm neonates has been associated with sepsis and a worse clinical outcome [33]. Kacsmarele et al. studied 47 preterm neonates weighing under 1250 g in 2013 and found a markedly lower HRV in neonates that experienced extubation failure. This test had a specificity and positive predictive value of close to 100%. However, this study suggested that more research was needed to consider HRV as a tool for assessing extubation readiness in neonates [34].

Several studies investigated the predictive ability of respiratory variability index (RVI) for successful extubation and found lower RVI in patients with extubation failure. In 2013, a review of 44 neonates showed that reduced RVI in preterm neonates could predict extubation failure. This study found that a combination of RVI and clinical response to SBT had high sensitivity and specificity relative to either alone in predicting successful extubation [35]. Shalish W. et al. in a prospective, multicenter study designed an automated tool that analyzed by MATLAB compiler for prediction extubation readiness in extremely preterm infants. the use of machine learning and automated RVI and HRV assessment methods in this tool can reduce extubation failure from 20% to less than 5%, and cost-effectivity of preventation of prolong ventilation. However, more research on these indexes, which use complex analyses over time, is needed to apply these techniques to clinical practice [36].

### Extubation checklists

Sur et al. published a review that provided checklists of criteria that a neonate should meet 24 hours before extubation. At the beginning of the shift, the physician should inform the shift coordinator of the potential for extubation. Steps may be taken, such as implementing a trial on CPAP, stopping feeding, SBT (optional), and changing to pressure support (optional) two hours before the planned extubation. CDP devices can be readied one hour before extubation. Nursing care and observation of the neonate for signs of increased work of breathing are performed 30 minutes before extubation, and finally, the neonate is extubated [37]. These protocols may reduce the stressful experience of extubation and reintubation, but there are no generally accepted guidelines for these checklists.

### Quality improvement (Q.I.) studies on the success of extubation

Prasad performed a quality improvement study in 2018 to improve successful extubation. His research aimed to reduce extubation failure. A Plan-Do-Check-Act (PDCA) cycle tool was implemented. The rate of reintubation following extubation decreased from 41.7% (pre-protocol period) to 23.8%. After data collection and brainstorming, the most likely causes of identified extubation failure were put into a fishbone diagram. The investigators determined that most causes of failure are due to human factors and method groups [38]. Nair et al. performed a quality-improved intervention to reduce unexpected extubation in neonates. They first identified SMART goals and started quality improvement interventions. This study shows that after implementing Q.I. interventions, there was a significant decrease ;approximately 80% in the unexpected extubation rates [39]. These studies also show that familiarity with Evidenced-based Practice for Improving Quality (EPIQ) can assist neonatologists in reducing unplanned extubations and improving extubation success.

### Post extubation management

After extubation, premature neonates may have inadequate respiratory drive and muscle strength to maintain functional residual capacity (FRC). In addition, the neonates' vocal cords may become edematous during intubation, preventing effective grunting for a variable period after extubation. The inability to oppose the vocal cords to generate endogenous distending pressure deprives the baby of a mechanism that preterm neonates naturally use to augment end-expiratory volume. For this reason, it is necessary to provide a continuous distending pressure (CDP) for all neonates immediately after extubation. After extubation, different types of CPAP have been used to provide this pressure for neonates, especially preterm neonates. Furthermore, other non-invasive modalities have been gradually employed to provide ventilatory support for neonates after extubation, such as non-invasive positive pressure ventilation (NIPPV) and humidified high-flow nasal cannula (HFNC). These latter modalities may not generate as much positive end-expiratory pressure as nasal CPAP.

It was previously believed that the pharyngeal pressure generated by HFNC is low and unstable, and HFNC cannot create the required CDP. Evidence has shown that failure following extubation with high-flow nasal cannulas is more significant than with traditional nasal CPAP. Heated humidified HFNC (HHHFNC) is associated with more apnea, bradycardia, and increased work of breathing after extubation [40, 41]. However, more recent studies have revealed contradictory results. Ramaswamy et al., in a systematic review of 33 studies and 4080 neonates, found that the effect of HFNC was similar to nasal CPAP and that it was the safest and most comfortable non-invasive modality for post-extubation with minimal nasal trauma and air leak compared to other methods [42]. Moreover, according to a Cochrane review regarding the administration of HHHFNC in preterm neonates, the effect of this modality on preventing treatment failure, CLD, and death was similar to other modalities with less nasal trauma and pneumothorax [43]. Several studies have supported these results; however, more evidence is required to support the use of HHHFNC for post-extubation care in neonates born before 28 weeks.

Synchronized and non-synchronized non-invasive positive pressure ventilation (NIPPV) are other modalities for respiratory support of neonates after extubation. Synchronized NIPPV can provide synchronized inflations with the baby's inspiration, known as SNIPPV.

Several studies have compared the efficacy of NIPPV and nasal CPAP after extubation in neonates. In 2017, a Cochrane review of 10 clinical trials comparing these two modalities showed that NIPPV effectively reduced extubation failure and reintubation but had no effect on the incidence of chronic lung disease and mortality rate. The meta-analysis demonstrated that the risk of extubation failure decreased statistically (typical RR 0.70, 95% CI 0.60 to 0.80; typical RD -0.13, 95% CI -0.17 to -0.08; NNTB 8, 95% CI 6 to 13; 10 trials, 1431 infants) and required re-intubation (typical RR 0.76, 95% CI 0.65 to 0.88; typical RD -0.10, 95% CI -0.15 to -0.05; NNTB 10, 95% CI 7 to 20; 10 trials, 1431 infants). Moreover, synchronized NIPPV was more effective than non-synchronized NIPPV or NIPPV provided by a bilevel device [44]. The findings of this review were confirmed by studies in 2020 and 2021 [42, 45]. Other studies that compared high CPAP (between 9 and 14 cmH2O) and NIPPV showed no difference in outcome and complications between these two modalities. A multicenter clinical trial study of 1440 neonates in 2019 showed that high CPAP could have a similar effect to NIPPV and non-invasive HFOV (NHFOV) in reducing the risk of extubation failure [46]. However, there are controversies about the use of high CPAP since it has been associated with complications such as air trapping and pulmonary air leaks. NIPPV has been reported to be more effective than CPAP in 2 studies, but it should preferably be synchronized [45, 46]. Noninvasive Neurally Adjusted Ventilatory Assist (NAVA) and NHFOV are other non-invasive ventilatory support modalities used after extubation in neonates. In cases where hypercapnia complicates extubation, NHFOV may be more effective than NCPAP, but there are few studies investigating this in neonates.

Apart from providing a proper CDP, other factors during and after extubation may also play a role in the success rate.

### Permissive hypercapnia

Permissive hypercapnia (P.H.) is an effective strategy that permits relatively high levels of PCO2 to decrease the incidence of VILI. Several studies have evaluated the benefits of P.H. during the weaning process to accelerate extubation. It is hypothesized that preterm neonates exposed to hypercapnia have a better respiratory drive, and studies have shown fewer days of invasive mechanical ventilation and lower rates of BPD [47, 48]. However, although mild permissive hypercapnia has been considered safe in randomized controlled trials (RCTs) and other studies, it has been reported to be associated with little clinical benefit in preventing extubation failure [49]. The current PaCO2 values used as cut-offs are different based on specific disease states, but several studies defined P.H. as PaCO2 levels between 45 and 65 mmHg as long as the pH remained 7.20 or higher. However, the safe limits of P.H. in the early days of life, when the risk of intraventricular hemorrhage (IVH) is the highest, and later in the chronic phase of BPD are not yet clear [50].

### Permissive hypoxemia

Oxygen supplementation to preterm neonates is a common intervention. The existing knowledge on the range of optimal oxygen saturation at different gestational ages and postnatal ages and its effect on the weaning process is still debated. In the BOOST study, neonates in the high O2 saturation range (91-95%) received oxygen longer than the low O2 group (85-89%); moreover, they were more O2 dependent at 36 weeks' postmenstrual age (PMA) and received higher rates of home O2 therapy without significant improvement in growth and neurodevelopment. Furthermore, the BOOST-II study conducted in the U.K. and Australia found no marked difference in mortality and disability between the two groups [51, 52]. In the Neonatal Oxygenation Prospective Meta-analysis (NeOProM), a total of 4965 premature infants <28 weeks gestation were studied within the first 24 hours after birth and found that the high O2 saturation range increased the risk of ROP but decreased the incidence of severe NEC and mortality [53, 54]. In 2020, Youstina Hanna et al. studied 145 preterm infants and reported an 18% absolute reduction in BPD incidence in the higher saturation target group (90 to 95%) compared with the lower oxygen saturation target group (88 to 92%). Due to the sigmoid shape of the oxyhemoglobin dissociation curve, there are more significant fluctuations in the partial pressure of arterial oxygen in the low O2 saturation range, thus causing the weaning of oxygen to be delayed in this group [55].

In summary, according to the results of several studies, a lower target SPO2 minimizes the severity and prevalence of ROP and decreases the need for ventilatory support and oxygen supplementation despite a slight statistically significant increase in mortality. Further research is necessary to determine the effectiveness of permissive hypoxemia in improving pulmonary outcomes. However, many investigators believe that oxygen saturation levels below 88% should be avoided in preterm neonates.

### Caffeine

Several studies have evaluated the effect of caffeine on preterm neonates. In the CAP study, caffeine was administered to neonates weighing 500-1250 g in the first ten days of life. More than half of the caffeine group neonates received treatment while on mechanical ventilation. The results revealed that extubation was earlier in 33% of cases, and post-extubation apnea was reduced by 20% in the caffeine group.

Secondary data analysis showed that neonates in the caffeine group had shorter lengths of time on mechanical ventilation, CPAP, oxygen therapy and lower BPD rates. Subsequent studies have revealed that earlier caffeine administration was associated with faster weaning. Although caffeine administration is safe and effective for weaning in preterm neonates weighing under 1250 g, these findings were secondary analysis results and should be interpreted cautiously [56, 57].

In 2010, a Cochrane systematic review found that caffeine administration before extubation increased the chance of successful extubation and improved the developmental outcome of neonates treated with this medication. Extubation failure is reduced within one week following treatment with Methylxanthines (summary RR 0.48, 95%CI 0.32 to 0.71; summary RD -0.27, 95%CI -0.39 to -0.15; NNT 4, 95%CI 3 to 7; six trials, 172 infants) [58]. Studies conducted in recent years have mainly focused on the dose and timing of caffeine administration. A clinical trial study published in 2018 showed that caffeine should be initiated with caution in the first five days of life since early initiation had no effect on accelerating weaning and even increased mortality. Successful extubation age after early caffeine treatment did not differ with control groups (median, 24 days; IQR, 10-41 days VS, median, 20 days; IQR, 9-43 days; P = .7) [59]. Another clinical trial published in 2021 reported different results. According to this study, caffeine administration at the initiation of mechanical ventilation accelerated extubation without causing complications [60]. This variation in results is also seen in other cohort studies, and it seems that clinical trials with larger studied populations and longer follow-up times may help determine the optimal use of caffeine. Petter Brattström et al. performed a systematic review in 2019 and found that using a higher maintenance dose of caffeine was associated with lower mortality and BPD rates [61]. These results have been confirmed in other studies [62]. Based on multiple studies, caffeine administration appears indicated, especially in very low weight preterm infants at risk of apnea before extubation.

### Postnatal corticosteroids

Several studies have investigated the effectiveness of postnatal corticosteroid administration on premature neonates with RDS. The results showed the effect of corticosteroids on facilitating extubation, BPD-free survival, and other outcomes [63–66]. According to these studies, treatment with low-dose hydrocortisone in the first two weeks of life increased BPD-free survival, especially in neonates born with prenatal inflammation.

Studies showed that high-dose dexamethasone (0.5 mg/kg/day) reduced the prevalence of BPD but was associated with several short-term and long-term complications, including neurodevelopmental delays. In general, high-dose corticosteroid therapy is not better than low-dose treatment.

The AAP, in a Policy Statement in 2010, advised: first, a high dose of dexamethasone (about 0.5 mg/kg/day) has been shown to reduce the prevalence of BPD but was accompanied by several long and short-term complications. Second, low-dose dexamethasone (less than 0.2 mg/kg/day) facilitates extubation without high-dose therapy's long and short-term complications. Third: treatment with low-dose hydrocortisone (1 mg/kg/day) in the first two weeks of life improved BPD-free survival, especially in neonates born with prenatal inflammation without an increase in neurodevelopmental delays. However, physicians should be aware of the increased risk of intestinal perforation associated with the concomitant use of prostaglandin inhibitors. Fourth there is insufficient data regarding treatment with high-dose hydrocortisone.

Twenty-one RCTs of postnatal corticosteroid use analyzed in a review article showed an unfavorable risk/benefit ratio in neonates with mild disease. In contrast, the outcome of late corticosteroid administration was favorable for cases where the baby cannot be weaned from mechanical ventilation [67].

Several studies have investigated the timing of corticosteroid administration (early administration within the first seven days of life versus later administration). In these studies, administration after the first week effectively facilitated extubation and reduced BPD. This protocol caused fewer neurodevelopmental complications; despite accelerating extubation with corticosteroid administration in the first eight days, it was associated with several short-term difficulties, including G.I. bleeding, intestinal perforation, hyperglycemia, hypertension, cardiomyopathy, and short-term growth disorder. Moreover, long-term complications such as neurodevelopmental disorders and cerebral palsy were more common with this treatment method [63, 67–69].

Corticosteroids can also be used during the weaning process and after extubation to treat stridor, a relatively common occurrence after neonatal extubation that increases the risk of extubation failure in extremely low birth weight (ELBW) neonates. The risk factors for stridor include prolonged mechanical ventilation, multiple intubations, and history of extubation failure. While a Cochrane review in 2008 showed that corticosteroids had no confirmed role in the prevention or treatment of post-extubation stridor in neonates and children [70], subsequent evaluations revealed that corticosteroids were an acceptable treatment in post-extubation stridor [71].

Nonetheless, although corticosteroids, even at lower doses, can be effective in the weaning process, risk-benefit assessments should be done, and the physician should use clinical judgment to maintain the balance between positive effects on lowering the incidence of BPD and side effects in each patient. The U.S. National Institute of Child Health has developed a BPD outcome calculator which may assist in predicting which infants may benefit from postnatal steroids with a sensitivity of 84%-92% and 77%-80% specificity at risk more than 37% in 14 days [72].

If corticosteroid therapy is necessary, it should be prescribed for a short time, and the risks and benefits should be discussed with parents. Using inhaled, intra-nasal, or intra-tracheal corticosteroids is also acceptable, but more evidence is needed. The AAP advises against the ***routine*** use of corticosteroids in early-stage RDS to prevent BPD and shorten mechanical ventilation time [73].

### Diuretics

Several studies indicating improved lung mechanics following the administration of diuretics have resulted in the use of furosemide, thiazide, and spironolactone for neonates with BPD and/or RDS; however, little evidence supports their administration in preterm neonates [74, 75]. The use of diuretics in RDS or BPD has been based on the hypothesis that these patients suffer from pulmonary interstitial edema resulting in increased fluid accumulation in the alveoli, and fluid restriction and short-term use of diuretics will benefit them. However, the effect of diuretics on the length of ventilatory support and oxygen supplementation, length of hospital stay, possible complications, and the long-term outcome has not been demonstrated in multiple trials.

The possible benefits of these drugs should be weighed against their known side effects before making routine administration decisions [76].

### Chest physiotherapy

Mechanical ventilation increases pulmonary secretions and causes atelectasis, which is associated with adverse outcomes in neonates. It is hypothesized that chest physiotherapy, including percussion and vibration before and after extubation, may facilitate the removal of these secretions and improve pulmonary function [77]. However, a Cochrane systematic review in 2010 showed that chest physiotherapy had no effect on reducing pulmonary secretions, and there was no evidence for its use. Moreover, this review did not refute or confirm complications such as IVH secondary to chest physiotherapy [78]. Chest physiotherapy may effectively prevent atelectasis, but the available data are inconclusive.

### Optimal nutritional support

The role of nutrition in facilitating extubation has not received adequate study. However, it seems logical that neonates should be provided with aggressive nutritional support during intubation to prepare them for the post-extubation phase.

### Developmentally supportive care

Rashwan's study on 62 mechanically ventilated neonates showed that the mechanically ventilated neonates placed in a side-lying position and neonates wrapped with a mother-scented simulated hand had significantly lower distress, neonatal pain, and higher mean SpO2 values. Implementing these techniques postulates that it would be easier to separate the baby from the device (i.e., ventilator) by improving oxygenation [79]. More studies on these and other supportive care are needed.

### Extubation failure

Prolonged mechanical ventilation strongly correlates with increased morbidity and mortality in preterm neonates, which is why neonatologists tend to wean neonates from mechanical ventilation as soon as possible. Most preterm neonates are easily extubated following short-term mechanical ventilation, but some experience reintubation for several reasons. It is unclear whether early weaning, failure, and reintubation directly worsen these babies' short-term or long-term outcomes. Extubation failure is defined as the need for reintubation after extubation within a predefined time, depending on the healthcare center criteria, that usually ranges between 2-7 days [80], which may be different according to the degree of prematurity. Reintubation due to non-respiratory causes such as sepsis and gastrointestinal problems during these seven days is rare. After seven days, reintubation is usually secondary to new pathologies unrelated to the neonate's condition immediately after extubation.

The prevalence of extubation failure ranges between 10-80% in preterm neonates depending on the demographic characteristics of the study population, age at extubation, length of time on mechanical ventilation before extubation, and post-extubation management. Several factors have been investigated as the possible causes of extubation failure. Factors such as respiratory muscle weakness, airway abnormalities, hemodynamically significant patent ductus arteriosus (PDA), poor respiratory control, lung injury, and nosocomial infection, especially ventilator-associated pneumonia (VAP), all may contribute to extubation failure. According to several studies, low G.A., i.e., birth before 26 weeks gestation, receiving mechanical ventilation for more than 10-14 days, low pH, higher PCO2 before extubation, extubation from high settings (high MAP and FiO2), and a low SPO2/FiO2 ratio increase the risk of failure. Other causes of extubation failure include inconsistent respiratory drive, poor respiratory pump, upper airway malacia, alveolar atelectasis, hemodynamic instability, hemodynamically significant PDA, glottic and subglottic edema, and residual lung injury such as BPD. Among the ventilator parameters, some investigators have noted a relationship between tidal volume before extubation and extubation failure [81–84].

Other causes of extubation failure include factors inherent to the NICU or its policies:

1. Inadequate training of personnel
2. Non-availability of respiratory therapists
3. Inadequate training of residents
4. Early extubation (especially in ELBW infants)
5. Lack of a protocol for initiation and termination of mechanical ventilation
6. Use of sedatives and muscle relaxants
7. Delay in obtaining chest radiography and/or arterial blood gases
8. Lack of proper post extubation CDP after extubation

Several studies evaluated the relationship between reintubation and mortality/morbidity in extremely premature neonates but found no direct cause-effect relationship between reintubation and BPD or death. However, reintubation increased the time of exposure to mechanical ventilation by 10-12 days, which was associated with both short and long-term complications [82, 85, 86].

Weaning from mechanical ventilation is not benign and disturbs the patient's physiology. It may also result in repeated attempts and failures to reintubate and the related complications, which may result in endotracheal tube malposition; trauma to the nose, glottis, trachea, and lungs; airway collapse; asphyxia, and infection. These complications may cause or aggravate neurological and cardiorespiratory disorders and lead to long-term respiratory and neurological disabilities. Reports of severe bradycardia and marked changes in blood pressure, oxygen saturation, and intracranial pressure during intubation confirm the importance of reducing the rate of extubation failure without causing an unnecessary increase in the length of time on mechanical ventilation.

To control the extubation process, caretakers should consider the following points in each NICU. There should be a single definition of extubation failure, and the rate of extubation failure and the respiratory/non-respiratory causes of each reintubation should be determined. Unplanned extubations with the need for reintubation should be tracked with efforts to decrease this rate per 100 ventilator days. Severe and dangerous post-extubation complications should be closely monitored and documented during the first 24 hours, including hemodynamic instability, severe or prolonged hypoxia, severe IVH, and death.

Finally, the decision regarding the extubation of neonates, especially preterm neonates, is complex and depends on several factors affecting the outcome. Despite extensive studies and research, the predictors and determinants of successful extubation are currently unclear. Unfortunately, the available tools cannot predict the appropriate extubation time for any specific baby. More studies and newer technologies are needed to improve the process of weaning and extubation in neonates. However, applying a single protocol in each center according to the available equipment and facilities and considering the clinical condition of each neonate can increase the chance of success and improve neonatal outcomes.

### Cost-effectiveness of early extubation

Sharma et al. reviewed the cost of a NICU stay published in various older studies. They found that cost varies based on the neonate's gestational age, level of care, and degree of illness, varying between 90 USD to 1250-2500 USD per day. The cost of NICU can be reduced with several interventions, including preventing invasive mechanical ventilation with the widespread use of CPAP. Regarding cost-effectiveness, CPAP reduces costs compared to NIPPV and MV [87].

## Conclusion

Clinicians should remember that the neonate's clinical comfort should be achieved with any change in ventilator setup. To experience a smooth weaning process, our protocol is to initially decrease FiO2 to 30% while SPO2 is kept between91%-95%. Based on the PaCO2 level, we reduce the tidal volume and rate. With improving oxygenation, PEEP is decreased. Caffeine is loaded, especially in very low weight preterm infants, and all sedatives are discontinued. Before weaning, the neonate may be placed on CPAP for 3-10min, and if there is no discomfort or increased work of breathing, the neonate is extubated, and NIPPV is administered. Extensive use of NIPPV after extubations helps early weaning resulting in a higher MAP than CPAP or HFNC alone. Other techniques, such as maintaining hand hygiene, preventing overcrowding in the NICU, using protocols to reduce infection rate (especially VAP), and aggressive nutritional support, may help improve overall respiratory outcomes.

## Data Availability

Data sharing is not applicable to this article as no datasets were generated or analysed during this study.

## Abbreviations

A.C: Assist-Control
ANS: Autonomic Nervous System
CDP: Continuous Distending Pressure
CPAP: Continuous positive airway pressure
PCV: Pressure Control Ventilation
ELBW: Extremely Low Birth Weight
ETT: Endo Tracheal Tube
GA: Gestational Age
GI: Gastro-Intestinal
HFV: High-Frequency Ventilation
HFOV: High-Frequency Oscillatory Ventilation
HHFNC: Humidified High Flow Nasal Cannula
HR: Heart Rate
HRV: Heart Rate Variability
IVH: IntraVentricular Hemorrhage
MAP: Mean Airway Pressure
MV: Mechanical Ventilation
NCPAP: Nasal Continuous Positive Airway Pressure
NHFO: Nasal High-Frequency Oscillation
NIPPV: Non-Invasive Positive Pressure Ventilation
PAW: Mean Airway Pressure
PEEP: Positive End Expiratory Pressure
PIP: Positive Inspiratory Pressure
PMA: Post Mesturation Age
P.S: Pressure Support
PTV: Pressure Targeted Ventilation
RDS: Respiratory Distress Syndrome
RR: Respiratory Rate
SBT: Spontaneous Breathing Trials
SIMV: Synchronized Intermittent Mandatory Ventilation
TTI: Tension-Time Index
VAP: Ventilator-Associated Pneumonia
VILI: Ventilator-Induced Lung Injury
VTV: Volume Targeted Ventilation

## References

[1] Walsh MC, Morris BH, Wrage LA, Vohr BR, Poole WK, Tyson JE (2005). Extremely low birth weight neonates with protracted ventilation: mortality and 18-month neurodevelopmental outcomes. J Pediatr..

[2] Stoll BJ, Hansen NI, Bell EF, Shankaran S, Laptook AR, Walsh MC (2010). Neonatal outcomes of extremely preterm infants from the NICHD Neonatal Research Network. Pediatr..

[3] Miller JD, Carlo WA (2008). Pulmonary complications of mechanical ventilation in neonates. Clin Perinatol.

[4] Vliegenthart RJ, Van Kaam AH, Aarnoudse-Moens CS, Van Wassenaer AG, Onland W (2019). Duration of mechanical ventilation and neurodevelopment in preterm infants. Arch Dis Childhood-Fetal Neonatal Ed.

[5] Choi Y-B, Lee J, Park J, Jun YH (2018). Impact of prolonged mechanical ventilation in very low birth weight infants: results from a national cohort study. J Pediatr..

[6] MacIntyre NR (2001). Evidence-based guidelines for weaning and discontinuing ventilatory support: a collective task force facilitated by the American College of Chest Physicians; the American Association for Respiratory Care; and the American College of Critical Care Medicine. Chest..

[7] Hermeto F, Bottino MN, Vaillancourt K, Sant'Anna GM (2009). Implementation of a respiratory therapist-driven protocol for neonatal ventilation: impact on the premature population. Pediatrics..

[8] Blackwood B, Alderdice F, Burns K, Cardwell C, Lavery G, O'Halloran P. Use of weaning protocols for reducing duration of mechanical ventilation in critically ill adult patients: Cochrane systematic review and meta-analysis. Bmj. 2011:342. 10.1136/bmj.c7237 PMC3020589 21233157

[9] Bol B, van Zanten H, Wielenga J, Vd Hoogen A, Mansvelt P, Blackwood B (2020). Protocolized versus nonprotocolized weaning to reduce the duration of invasive mechanical weaning in neonates: a systematic review of all types of studies. J Perinatal Neonatal Nurs.

[10] Chakkarapani AA, Adappa R, Ali SKM, Gupta S, Soni NB, Chicoine L, et al. “Current concepts in assisted mechanical ventilation in the neonate”-Part 2: Understanding various modes of mechanical ventilation and recommendations for individualized disease-based approach in neonates. Int J Pediatr Adolesc Med. 2020. 10.1016/j.ijpam.2020.11.002 PMC7729247 33319021

[11] Kadivar M, Sangsari R, Soltanalian H. Clinical Application of Neurally Adjusted Ventilatory Assist in Neonates with Respiratory Distress: A Systematic Review. J Compr Ped. 10(2):e62634.

[12] Klingenberg C, Wheeler KI, McCallion N, Morley CJ, Davis PG. Volume-targeted versus pressure-limited ventilation in neonates. Cochrane Database Syst Rev. 2017;(10). 10.1002/14651858.CD003666.pub4 PMC6485452 29039883

[13] Keszler M (2019). Volume-targeted ventilation: one size does not fit all. Evidence-based recommendations for successful use. Arch Dis Childhood-Fetal Neonatal Ed.

[14] Dassios T, Williams E, Ambulkar H, Shetty S, Hickey A, Greenough A (2020). Tidal volumes and outcome of extubation in mechanically ventilated premature infants. Am J Perinatol.

[15] Gupta S, Janakiraman S (2018). Volume ventilation in neonates. Paediatr Child Health.

[16] Clark RH, Null DM, Gerstmann DR, deLemos RA (1992). Prospective randomized comparison of high-frequency oscillatory and conventional ventilation in respiratory distress syndrome. Pediatr.

[17] Courtney SE, Durand DJ, Asselin JM, Hudak ML, Aschner JL, Shoemaker CT (2002). High-frequency oscillatory ventilation versus conventional mechanical ventilation for very-low-birth-weight infants. New England J Med.

[18] Cools F, Offringa M, Askie LM. Elective high frequency oscillatory ventilation versus conventional ventilation for acute pulmonary dysfunction in preterm infants. Cochrane Database Syst Rev. 2015;3. 10.1002/14651858.CD000104.pub4 PMC10711725 25785789

[19] Masry A, Nimeri NAMA, Koobar O, Hammoudeh S, Chandra P, Elmalik EE, Khalil AM, Mohammed N, Mahmoud NAM, Langtree LJ, Bayoumi MAA (2021). Reintubation rates after extubation to different non-invasive ventilation modes in preterm infants. BMC Pediatr..

[20] Chawla S, Natarajan G, Shankaran S, Carper B, Brion LP, Keszler M (2017). Markers of successful extubation in extremely preterm infants, and morbidity after failed extubation. The Journal of pediatrics..

[21] Shalish W, Latremouille S, Papenburg J, Sant’Anna GM. (2019). Predictors of extubation readiness in preterm infants: a systematic review and meta-analysis. Arch Dis Childhood-Fetal Neonatal Edition..

[22] Currie A, Patel D-S, Rafferty GF, Greenough A (2011). Prediction of extubation outcome in infants using the tension time index. Arch Dis Childhood-Fetal Neonatal Ed.

[23] Bhat P, Peacock JL, Rafferty GF, Hannam S, Greenough A (2016). Prediction of infant extubation outcomes using the tension-time index. Arch Dis Childhood-Fetal Neonatal Ed.

[24] Shalish W, Kanbar L, Kovacs L, Chawla S, Keszler M, Rao S (2020). Assessment of extubation readiness using spontaneous breathing trials in extremely preterm neonates. JAMA pediatrics..

[25] Mukerji A, Razak A, Aggarwal A, Jacobi E, Musa M, Alwahab Z (2020). Early versus delayed extubation in extremely preterm neonates: a retrospective cohort study. J Perinatol.

[26] Robbins M, Trittmann J, Martin E, Reber KM, Nelin L, Shepherd E (2015). Early extubation attempts reduce length of stay in extremely preterm infants even if re-intubation is necessary. J Neonatal-Perinatal Med.

[27] Berger J, Mehta P, Bucholz E, Dziura J, Bhandari V (2014). Impact of early extubation and reintubation on the incidence of bronchopulmonary dysplasia in neonates. Am J Perinatol.

[28] Ouellette DR, Patel S, Girard TD, Morris PE, Schmidt GA, Truwit JD (2017). Liberation from mechanical ventilation in critically ill adults: an official American College of Chest Physicians/American Thoracic Society clinical practice guideline: inspiratory pressure augmentation during spontaneous breathing trials, protocols minimizing sedation, and non-invasive ventilation immediately after extubation. Chest..

[29] Teixeira RF, Carvalho ACA, de Araujo RD, Veloso FCS, Kassar SB, Medeiros AMC (2021). Spontaneous breathing trials in preterm infants: systematic review and meta-analysis. Respiratory Care..

[30] Janjindamai W, Pasee S, Thatrimontrichai A (2017). The optimal predictors of readiness for extubation in low birth weight infants. J Med Assoc Thai..

[31] Nakato AM, de Fc RD, Simão AC, Da Silva RP, Nohama P (2021). Impact of spontaneous breathing trials in cardiorespiratory stability of preterm infants. Respiratory Care..

[32] Gillespie LM, White SD, Sinha SK (2003). Donn S.MUsefulness of the Minute Ventilation Test in Predicting Successful Extubation in Newborn Infants: A Randomized Controlled Trial. J Perinatol.

[33] Cardoso S, Silva MJ, Guimarães H (2017). Autonomic nervous system in newborns: a review based on heart rate variability. Child's Nervous Syst.

[34] Kaczmarek J, Chawla S, Marchica C, Dwaihy M, Grundy L, Sant'Anna GM (2013). Heart rate variability and extubation readiness in extremely preterm infants. Neonatology..

[35] Kaczmarek J, Kamlin COF, Morley CJ, Davis PG, Sant'Anna GM (2013). Variability of respiratory parameters and extubation readiness in ventilated neonates. Arch Dis Childhood-Fetal Neonatal Ed.

[36] Shalish W, Kanbar LJ, Rao S, Robles-Rubio CA, Kovacs L, Chawla S (2017). Prediction of extubation readiness in extremely preterm infants by the automated analysis of cardiorespiratory behavior: study protocol. BMC pediatrics..

[37] Sur A, Paria A. Weaning of invasive ventilation in the neonatal intensive care: Towards standardising practice: A narrative review. J Paediatrics Child Health. 2022;58(6). 10.1111/jpc.15995 35474623

[38] Prasad R, Mishra AK (2019). Improvement in Successful Extubation in Newborns After a Protocol-driven Approach: A Quality Improvement Initiative. Indian Pediatr..

[39] Nair V, Smith H (2020). Phased Quality Improvement Interventions in Reducing Unplanned Extubation in the Neonatal ICU. Respir Care..

[40] Wilkinson D, Andersen C, Smith K, Holberton J (2008). Pharyngeal pressure with high-flow nasal cannulae in premature infants. J Perinatol.

[41] Wilkinson D, Anderson C, O'Donnell C, De Paoli A (2011). High Flow nasal cannula for respiratory support in preterm infants (Review) The Cochrane Collaboration. Cochrane Library..

[42] Ramaswamy VV, Bandyopadhyay T, Nanda D, Bandiya P, More K, Oommen VI (2020). efficacy of non-invasive respiratory support modes as postextubation respiratory support in preterm neonates: A systematic review and network meta-analysis. Pediatr Pulmonol.

[43] Wilkinson D, Andersen C, O'Donnell CP, De Paoli AG, Manley BJ. High flow nasal cannula for respiratory support in preterm infants. Cochrane Database Syst Rev. 2016;2. 10.1002/14651858.CD006405.pub3 PMC9371597 26899543

[44] Lemyre B, Davis PG, De Paoli AG, Kirpalani H. Nasal intermittent positive pressure ventilation (NIPPV) versus nasal continuous positive airway pressure (NCPAP) for preterm neonates after extubation. Cochrane Database Syst Rev. 2017;2. 10.1002/14651858.CD003212.pub3 PMC6464652 28146296

[45] Sweet DG, Carnielli V, Greisen G, Hallman M, Ozek E, Te Pas A (2019). European consensus guidelines on the management of respiratory distress syndrome–2019 update. Neonatol.

[46] Shi Y, De Luca D (2019). Continuous positive airway pressure (CPAP) vs non-invasive positive pressure ventilation (NIPPV) vs non-invasive high frequency oscillation ventilation (NHFOV) as post-extubation support in preterm neonates: protocol for an assessor-blinded, multicenter, randomized controlled trial. BMC pediatrics..

[47] Thome UH, Ambalavanan N (2009). Permissive hypercapnia to decrease lung injury in ventilated preterm neonates. Seminars Fetal Neonatal Med.

[48] Brown MK, Poeltler DM, Hassen KO, Lazarus DV, Brown VK, Stout JJ (2018). incidence of hypocapnia, hypercapnia, and acidosis and the associated risk of adverse events in preterm neonates. Respiratory Care.

[49] Thome UH, Genzel-Boroviczeny O, Bohnhorst B, Schmid M, Fuchs H, Rohde O (2015). Permissive hypercapnia in extremely low birthweight infants (PHELBI): a randomised controlled multicentre trial. Lancet Respiratory Med.

[50] Wong SK, Chim M, Allen J, Butler A, Tyrrell J, Hurley T, et al. Carbon dioxide levels in neonates: what are safe parameters? Pediatr Res. 2021;1-8. 10.1038/s41390-021-01473-y PMC9122818 34230621

[51] Australia B-I, Groups UKC (2016). Outcomes of two trials of oxygen-saturation targets in preterm infants. New England J Med.

[52] Askie LM, Henderson-Smart DJ, Irwig L, Simpson JM (2003). Oxygen-saturation targets and outcomes in extremely preterm infants. New England J Med.

[53] Schmidt B, Whyte R. K. Oxygen saturation target ranges and alarm settings in the NICU: What have we learnt from the neonatal oxygenation prospective meta-analysis (NeOProM)? Seminars Fetal Neonatal Med 2020;25( 2): 101080 10.1016/j.siny.2020.101080 31983671

[54] Askie LM, Darlow BA, Finer N (2018). Association Between Oxygen Saturation Targeting and Death or Disability in Extremely Preterm Infants in the Neonatal Oxygenation Prospective Meta-analysis Collaboration. JAMA..

[55] Hanna Y, Laliberté C, Ben Fadel N, Lemyre B, Thébaud B, Barrowman N (2020). effect of oxygen saturation targets on the incidence of bronchopulmonary dysplasia and duration of respiratory supports in extremely preterm infants. Paediatr Child Health..

[56] Schmidt B, Roberts RS, Davis P, Doyle LW, Barrington KJ, Ohlsson A (2006). Caffeine therapy for apnea of prematurity. New England J Med.

[57] Davis PG, Schmidt B, Roberts RS, Doyle LW, Asztalos E, Haslam R (2010). Caffeine for Apnea of Prematurity trial: benefits may vary in subgroups. J Pediatr.

[58] Henderson-Smart DJ, Davis PG. Prophylactic methylxanthines for endotracheal extubation in preterm infants. Cochrane Database Syst Rev. 2010;12. 10.1002/14651858.CD000139.pub2 PMC12132029 21154342

[59] Amaro CM, Bello JA, Jain D, Ramnath A, D'Ugard C, Vanbuskirk S (2018). Early caffeine and weaning from mechanical ventilation in preterm infants: a randomized, placebo-controlled trial. J Pediatr.

[60] Awad HA, Tawfik aA, Ibrahim MJ, Hesham B. (2021). Effect of very early use of caffeine citrate in preterm neonates needing respiratory support. QJM: An Int J Med.

[61] Brattström P, Russo C, Ley D, Bruschettini M (2019). High-versus low-dose caffeine in preterm infants: a systematic review and meta-analysis. Acta paediatrica..

[62] Chen J, Jin L, Chen X. Efficacy and safety of different maintenance doses of caffeine citrate for treatment of apnea in premature infants: a systematic review and meta-analysis. BioMed research international. 2018:9061234. 10.1155/2018/9061234 PMC6323495 30671477

[63] Kurtom W, Schmidt A, Jain D, Vanbuskirk S, Schott A, Bancalari E, et al. efficacy of late postnatal dexamethasone on weaning from invasive mechanical ventilation in extreme premature infants. J Perinatol. 2021;1-5. 10.1038/s41372-021-01108-4 PMC8161346 34050243

[64] Tanney K, Davis J, Halliday HL, Sweet DG (2011). Extremely low-dose dexamethasone to facilitate extubation in mechanically ventilated preterm babies. Neonatology..

[65] Doyle LW, Ehrenkranz RA, Halliday HL (2010). Dexamethasone treatment after the first week of life for bronchopulmonary dysplasia in preterm infants: a systematic review. Neonatology..

[66] Filippone M, Nardo D, Bonadies L, Salvadori S, Baraldi E (2019). Update on postnatal corticosteroids to prevent or treat bronchopulmonary dysplasia. Am J Perinatol.

[67] Doyle LW, Ehrenkranz RA, Halliday HL. Late (> 7 days) postnatal corticosteroids for chronic lung disease in preterm infants. Coch Database Syst Rev. 2014(5(. 10.1002/14651858.CD001145.pub3 24825542

[68] Doyle LW, Cheong JL, Ehrenkranz RA, Halliday HL. Early (< 8 days) systemic postnatal corticosteroids for prevention of bronchopulmonary dysplasia in preterm infants. Coch Database Syst Rev. 2017;10. 10.1002/14651858.CD001145.pub4 PMC6485440 29063594

[69] Lemyre B, Dunn M, Thebaud B (2020). Postnatal corticosteroids to prevent or treat bronchopulmonary dysplasia in preterm infants. Paediatr Child Health.

[70] Markovitz B, Randolph A, Khemani RG (2008). Cochrane review: Corticosteroids for the prevention and treatment of post-extubation stridor in neonates, children and adults. Evidence-Based Child Health: A Cochrane Review Journal..

[71] Kimura S, Ahn JB, Takahashi M, Kwon S, Papatheodorou S (2020). Effectiveness of corticosteroids for post-extubation stridor and extubation failure in pediatric patients: a systematic review and meta-analysis. Ann Intens Care..

[72] Cuna A, Liu C, Govindarajan S, Queen M, Dai H, Truog WE (2018). Usefulness of an Online Risk Estimator for Bronchopulmonary Dysplasia in Predicting Corticosteroid Treatment in Infants Born Preterm. J Pediatr..

[73] Committee on Fetus and Newborn (2002). Postnatal corticosteroids to treat or prevent chronic lung disease in preterm infants. Pediatrics..

[74] Cotton R, Suarez S, Reese J (2012). Unexpected extra-renal effects of loop diuretics in the preterm neonate. Acta Paediatrica..

[75] Stewart A, Brion LP, Soll R. Diuretics for respiratory distress syndrome in preterm infants. Coch Database Syst Rev. 2011;12. 10.1002/14651858.CD001454.pub3 PMC7055205 22161366

[76] Blaisdell CJ, Troendle J, Zajicek A, Chougnet C, Greenberg JM, Hardie W (2018). Acute responses to diuretic therapy in extremely low gestational age newborns: results from the prematurity and respiratory outcomes program cohort study. J Pediatr..

[77] Ferguson KN, Roberts CT, Manley BJ, Davis PG (2017). Interventions to improve rates of successful extubation in preterm infants: a systematic review and meta-analysis. JAMA pediatrics..

[78] Hough JL, Flenady V, Johnston L, Woodgate PG (2010). Cochrane review: Chest physiotherapy for reducing respiratory morbidity in infants requiring ventilatory support. Evidence-Based Child Health: A Cochrane Rev J.

[79] Rashwan ZI, Khamis GM (2021). Does mother scented simulated hand promote comfort reduce pain, and distress among mechanically ventilated preterm neonates during invasive procedures?. J Health Sci.

[80] Giaccone A, Jensen E, Davis P, Schmidt B (2014). Definitions of extubation success in very premature infants: a systematic review. Arch Dise Childhood-Fetal Neonatal Ed.

[81] Kidman AM, Manley BJ, Boland RA, Davis PG, Bhatia R (2021). Predictors and outcomes of extubation failure in extremely preterm infants. J Paediatr Child Health..

[82] Manley BJ, Doyle LW, Owen LS, Davis PG (2016). Extubating extremely preterm infants: predictors of success and outcomes following failure. J Pediatr.

[83] Dassios T, Kaltsogianni O, Greenough A (2017). Relaxation rate of the respiratory muscles and prediction of extubation outcome in prematurely born infants. Neonatology..

[84] Gupta D, Greenberg RG, Sharma A, Natarajan G, Cotten M, Thomas R (2019). A predictive model for extubation readiness in extremely preterm infants. J Perinatol.

[85] Shalish W, Kanbar L, Keszler M, Chawla S, Kovacs L, Rao S (2018). Patterns of reintubation in extremely preterm infants: a longitudinal cohort study. Pediatr Res.

[86] Jensen EA, DeMauro SB, Kornhauser M, Aghai ZH, Greenspan JS, Dysart KC (2015). Effects of multiple ventilation courses and duration of mechanical ventilation on respiratory outcomes in extremely low-birth-weight infants. JAMA pediatrics..

[87] Sharma D, Murki S (2021). Making neonatal intensive care: cost effective. J Matern Fetal Neonatal Med..

